# A Force-Sensor-Based Method to Eliminate Deformation of Large Crankshafts during Measurements of Their Geometric Condition

**DOI:** 10.3390/s19163507

**Published:** 2019-08-10

**Authors:** Krzysztof Nozdrzykowski, Leszek Chybowski

**Affiliations:** 1 Institute of Basic Technical Sciences, Faculty of Marine Engineering, Maritime University of Szczecin, Willowa 2-4, 71-650 Szczecin, Poland; 2 Institute of Marine Propulsion Plants Operation Faculty of Marine Engineering, Maritime University of Szczecin, Waly Chrobrego 1-2, 70-500 Szczecin, Poland

**Keywords:** large-size crankshafts, metrology, geometry measurements, force sensor, elimination of deformation, active measurement system

## Abstract

This article describes an innovative method for eliminating deformation in large crankshafts during measurement of their geometric condition. The currently available techniques for measuring crankshaft geometry are introduced and classified according to their applicability and the method of measurement. The drawbacks of the methods have been identified and a solution to these problems has been proposed. The influence of the rigid support of a shaft on its deformation, and thus on the reduction in the accuracy of crankshaft geometry measurements, has also been investigated. The concept and main versions of the proposed measuring system with active compensation for shaft deflection, by means of actuators cooperating with force transducers monitoring the deflection of individual crank journals of a crankshaft being measured, have been presented and the flexible support control system has also been described. The problems relating to the operation of the control system have been furnished along with a way to solve them, including the issue of noise reduction in the signal from the force transducer and the influence of the controller parameters on the operation of the flexible support. The computer system that controls the flexible supports has been briefly characterized, and the performance of the prototype system and the model reference system has been compared. The results have shown that the system is able to effectively eliminate the deflection and elastic deformation of the crankshaft under the influence of its own weight.

## 1. Introduction

A characteristic feature of machinery and equipment in ships’ engine rooms is the common occurrence of large-size elements. In this group of machine elements a specific group of parts, characterized by low and variable rigidity and high susceptibility to flexural deformation, can be distinguished. This group includes the crankshafts of ships’ engines, the manufacturing precision of which affects the correct operation of the crank-and-piston system to a large extent and thereby that of the entire engine. The cost of such a crankshaft accounts for approximately 20 ÷ 25% of the total cost of a ship’s engine.

The criterion for belonging to a group of large elements has not been clearly defined. The authors have proposed the adoption of a definition where machine parts that are larger than 3150 mm qualify for this group based on Humienny and Adamczak [1,2]. However, it should be stressed that in order to fully characterise this group of machine parts, additional criteria, i.e., rigidity and shape, would be required. With these additional criteria having been introduced, large crankshafts would be considered to be those where the length-to-diameter ratio (L/d) is greater than 12 ÷ 15, whereas the shape factor α_k_ determining the nature of cross-sectional changes may take on significant values α_k_ ˃ 1 (for a straight shaft with a constant diameter, affected by no sudden changes in cross-section α_k_ = 1) [3,4]. For a crankshaft, this coefficient also depends on other design details such as the location of the main journals and crank pins, their diameters, drive ratio, radii of their webs, angular arrangement, lubrication holes and grooves. With these structural features taken into account, the shape factor for specific shafts can be described by a complex mathematical relationship. These issues were tackled in works of Lang, Al-Azirjawi, Bulatović, Cao, Xue, Bai, Li, Dai and Gao et al. [5,6,7,8,9,10,11].

## 2. Overview of Crankshaft Geometry Measurement Systems

After reviewing the methods and techniques used to measure deflection in the shape and location of crankshaft axes, it can be stated that a decisive factor in this respect is the size of the shafts. An overview of the methods and techniques used to measure shafts, broken down by their dimensions and the position of an axis during measurement, has been presented in Table 1.

These methods can be subdivided into the following: non-reference methods consisting of the measurement of the radius changes and the reference methods consisting of the evaluation of the position of the points of the profile being measured in relation to one or more different points of that profile. However, the advancement in measurement technology has provided an impulse to single out one more group of measurement methods, i.e., a measurement based on an object’s image, which include scanning and photometry.

In the case of small-sized shafts (high-speed engines with a nominal speed above 1500 rpm), reference measurements in a device with centring cones are most common [12]. Such a measurement is possible only if centre holes have been previously made in the shaft being measured and, taking into account the load-bearing capacity of the locating centres, the shaft has small dimensions and a low weight. More advanced measuring systems of this type are coupled with a computer and make the digital measurement of the roundness profile possible. Such systems were proposed by Adamczak, Kim, Lee, Nwagboso and Novac [12,13,14,15,16]. The measurement error of this method depends on the manufacturing accuracy and the type of centre holes, as well as the manufacturing accuracy of the centre device, the type of sensor used and the method and accuracy of the preparation of the results. Such solutions are especially used in workshop measurement techniques. The shaft’s axis is horizontal and, in the simplest solution, can be kept in this position using a universal centre device and a sensor set in a tripod.

For these type of shafts, as well as crankshafts of medium-speed engines (nominal speed of 450–1500 rpm), whose crankshafts are not large ones, non-reference measurement methods with a rotary table [17,18,19,20] or with a rotary spindle [18] are used. Such measurements may be carried out using specially adapted instruments or measuring machines.

In these cases, the position of the crankshafts’ axes is usually vertical. The measuring machines that are currently available enable full, comprehensive and quick assessment of the shafts’ geometric condition. In these instruments, the measuring tip of the sensor moves as the radius of the object being measured changes. The resulting signal, when processed and amplified, makes the recording of the contours measured possible in the form of a graph and therefore the determination of the contour’s evaluation parameters. Such instruments or measuring machines are offered by the world’s leading makers of measuring instruments, which include the following: Taylor Hobson Ltd. (Leicester, UK) offering the Talyrond 440 [17], Mahr GmbH (Göttingen, Germany) offering various solutions in machines measuring roundness and other quantities, including the PRIMAR GMX 600 measuring machine [18] and the MarForm MFK 600 [18], the JENOPTIC Hommel-Etamic Shaftscan 1030 [19], Perthen [18], and ADCOLE Co. (Marlborough, MA, USA) which stands out in the manufacture of specialized measuring machines for shafts, offering the ADCOLE 1200 measuring machine [18], which enables comprehensive and quick measurements of crankshafts that are between 500 to 3300 mm in length.

In most cases, these machines can measure shafts that are positioned vertically without any additional support. Instruments designed for measurements with non-reference methods are able to provide a high accuracy of measurement. Modern solutions of non-referenced measuring instruments, in which the use of computer technology is currently the standard, make it possible to eliminate a number of errors, especially systematic errors [12].

Non-reference measurements of shafts, even those with large dimensions, can also be carried out by coordinate measuring machines (CMM) [21,22,23,24]. This has been confirmed by the research conducted by Wieczorowski, Marciniak and others, the results of which are presented in the literature [21], the aim of which was to analyse the capability for measuring crankshafts using coordinate techniques, including the Leitz PMMG coordinate measuring machine [24].

Measuring instruments or machines performing non-reference measurements are accurate, but they are mainly used in laboratories, because they require specific measuring conditions, e.g., accurate and time-consuming centring and aligning of the measured objects. An additional limitation of the use of these instruments and machines is their high cost, because of which it cannot be economically justified to use them in the production of units in small batches or in overhaul conditions. It should also be stressed that the use of these machines will not eliminate the basic problems in fixing and supporting large and flaccid components [25,26,27,28,29,30,31].

Attempts have also recently been made to use modern measurement techniques in the measurement of large-size crankshafts. For example, the Tritop photometric system and the system based on the Atos Trident III optical scanner were used to coarsely quantify the suitability of a semi-finished product for the technological process of machining a 40-tonne forged crankshaft with a length of 13 m. The measurable effect of using such a measurement technique was the shortening of the routing, previously carried out by two employees by a traditional method that took up to 24 hours, to less than 9 hours and requiring only one employee [32]. However, due to the low accuracy, the use of these techniques is very limited.

In the conditions of overhaul docks and repair workshops, reference measurement methods are much more useful for measuring deviation and shape contours after fixing the measured object in V-blocks [12,33,34,35,36,37,38]. In the case of shafts intended for medium and high-speed engines (for generating sets, traction motors, etc.), four V-blocks are most often used, while for large shafts of low-speed engines (rated speed below 450 rpm), many more V-blocks are used to fix and support the shaft [38].

In this case, the measurement procedures call for a series of separate measurements (performed mostly with the use of universal measuring equipment), which include—in addition to the measurements of linear and angular dimensions, the radial and axial run-out (whip) of the journals—the primary measurement plays a role in the final criterion for assessing the correctness of the geometrical manufacture of a shaft. This measurement gauges the deformation of the crank webs, called the deflection measurement [11,39,40]. This measurement is considered as the final criterion for the assessment of the geometrical condition of the shaft, which results from the lack of the possibility of the direct measurement of the deviation of the journal axes’ shape and position. The assessment of the deviation is therefore based on indirect measurements. It is problematic to eliminate the effect of elastic deformation when the shaft is supported by several fixed, rigid V-blocks because of the preset deflection and the existing geometric deviation. This state means that the geometrical deviation conjugates with and interacts with the elastic deformation and it is virtually impossible to eliminate them.

The measurement procedures for large crankshafts show significant gaps with regard to comprehensive measurements of geometric deviation. They are based on measurements that have been used for a number of years and whose accuracy has not been adjusted to take into account the increasing manufacturing accuracy that is expected of modern crankshafts. The solution to the problem requires a multi-criteria approach [41,42,43] which has been presented below.

## 3. The Effect of the Support Method on Elastic Deformation in the Object Being Measured

The axes of large crankshafts that are supported at multiple points can be dislocated as a result of elastic deformation caused by their own weight. Due to this performance of the shaft, what happens during the measurement of geometric deviations of the main journals is that the journal axis is positioned diagonally in relation to the plane in which the roundness profile is measured and the axis of displacement of the sensor’s probe stylus does not pass through the centre of the measured profile. For this reason, the measured roundness profile will be distorted by the so-called apparent eccentricity and ovality [44]. To achieve accurate measurements it is necessary to select the appropriate conditions to support the shaft to eliminate deflection of the shaft under the dead weight [45,46,47,48,49].

**Figure 1:**
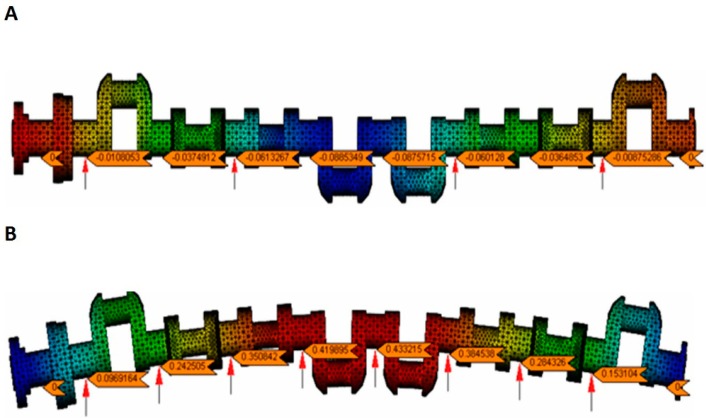
The deflection in the central part of the crankshaft assuming zero deflection at the outermost journals: (**A**) crankshaft rested on four rigid supports spaced evenly along the shaft; (**B**) the shaft rested on eight supports loaded with a uniform reaction force of 1080.5 N ensuring zero deflection at the outermost journals.

Analysis presented in the sources [49,50,51] have shown that only supporting selected main journals of a shaft—a common practice in measurements in manufacturing conditions or in repair (overhaul) workshops—resulted in significant deformation (Figure 1). Figure 1A shows the deflection of a crankshaft seated on four fixed, rigid supports spaced along the shaft, assuming zero deflection at the outermost journals. The deflection at the journals in the central part of the shaft ranged from −0.00875 to −0.0885 mm. The adoption of a support variant, where all the main journals in the central part of the shaft were mounted on fixed supports with the same support reaction forces, also did not eliminate the shaft’s deflection; such a case has been shown in Figure 1B. The deflection for this variant of support ranged from +0.00969 to +0.43320 mm. In both the presented case and the other analysed cases of supporting the shaft with fixed supports, there was significant elastic deformation of the shafts [50,51,52]. However, the simulation tests have shown that eliminating shaft deflection may provide support for all the shaft’s main journals with a set of supports that will exert variable reactive forces where the supports’ heads contact the journals. The values of the reaction forces should not only vary along the shaft, but also vary depending on the shaft’s rotation angle at the supports [44,49,50].

**Figure 2:**
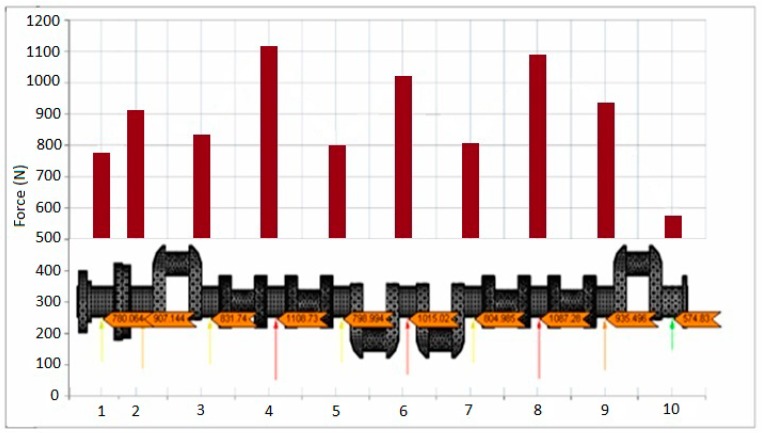
Distribution of forces ensuring zero deformation of the main journal of the crankshaft of a Buckau Wolf R8DV 136 engine for a shaft position of 0 °CA.

Figure 2 has shown example of the calculations of the required reaction forces at the individual main journals for the one of characteristic angular positions, for an object undergoing tests; namely the crankshaft of a Buckau Wolf R8DV136 ship main propulsion medium-speed engine, based on the NASTRAN FX 2010 (The MacNeal-Schwendler Corporation, Newport Beach, CA, USA) strength calculation software.

If there is no support under any of the main journals or if the reaction forces other than those required to eliminate any deflection are applied, significant deflection occurs and cannot be eliminated. Maintaining strictly defined positions of the supports, which exert precise reaction forces corresponding to these positions, eliminates totally any deflection of a shaft. If the preset forces are exerted and supports are wrongly positioned, then the effect will be that the shaft’s deflection will change significantly.

## 4. Measurement System with Controlled Exertion of the Supports’ Reaction Forces

**Figure 3:**
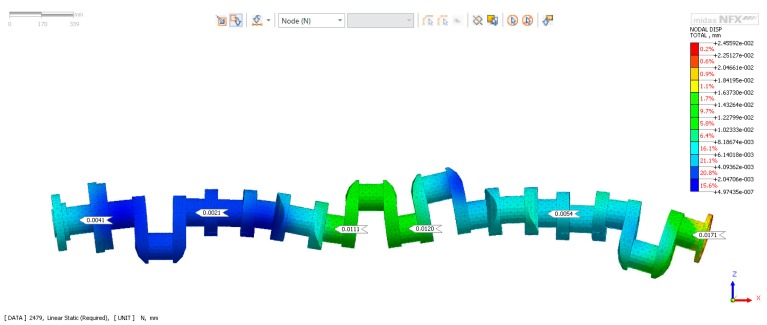
Deflection of the shaft supported by the four main journals, with reaction forces so selected that there was no deflection at journals 2, 4, 7, 9 counting from the timing gear side.

Supporting the shaft in a controlled manner makes a significant reduction in deflection possible, even if only selected main journals of a shaft are supported. Figure 3 presents the deflection of a shaft supported on the four main journals, as in the case shown in Figure 1A, with the values of the reaction forces (determined from FEM) analysis selected so that there was no deflection on the supported journals (numbers 2, 4, 7, 9 counting from the timing gear side).

**Figure 4:**
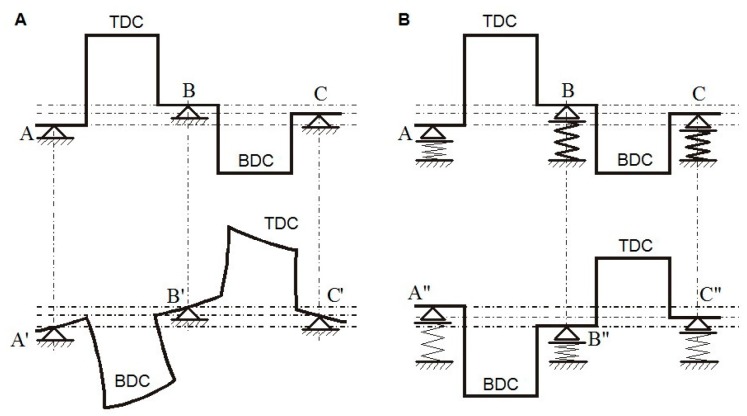
Representation of the deformation of crankshaft webs [45]: (**A**) rigid support of the shaft; (**B**) flexible support of the shaft.

It should also be stressed that if the coaxiality of the main journals is not maintained when the shaft rotates during the geometry measurements, then additional elastic deformation of the shaft will occur due to uncontrolled support reactions (Figure 4A). The results of the analysis that has been presented were the basis for the development of an innovative measurement system with the controlled exertion of reaction forces by the supports, based on the concept of the so-called flexible shaft support. The idea of an elastic support on a section of a shaft’s double crank has been presented in Figure 4B.

**Figure 5:**
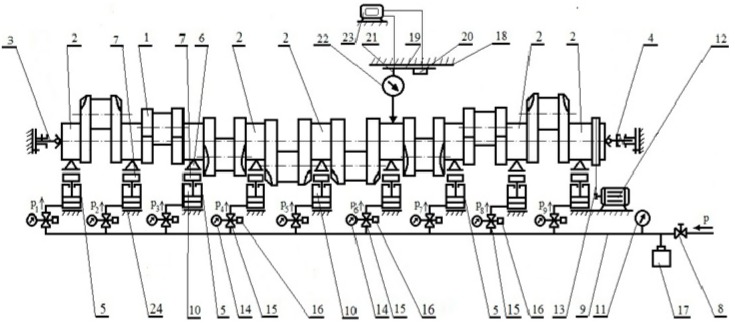
Diagram of the basic elements of the measurement system for a crankshaft fixed at the outer faces by means of centres [51].

The use of flexible supports makes it possible to virtually eliminate the elastic deflection of the shaft. Regardless of the possible geometrical deviations, the elastic supports that exert the preset reaction forces act as flexible elements that compensate for any possible elastic deflection of the shaft. Figure 5 is a diagram of the components and subassemblies of the proposed system for compensating for shaft deformation during geometry measurements [32,33]. Four primary elements that make up the entire system are the flexible support and shaft fixing system, the measuring system, the shaft rotation control system and the data processing and analysis system.

The elastic support system consists of a set of elastic supports (5), the number and arrangement of which depend on the number and arrangement of the main journals (2) of the crankshaft (1). These supports are equipped with self-adjusting rolling heads (6) consisting of V-blocks, which have all degrees of freedom, like the actuators (10) (driven by a specific medium). The flexibility of the resilient supports compensates for the vertical and horizontal deflection and displacement of the shaft. These supports are elements that exert a passive reaction force in order to eliminate elastic deformation of the measured object, and at the same time compensate for any displacement caused by the geometrical deformation of that object. The variable pressures *p_i_* and the corresponding forces in the actuators (10) of the relieving supports are continuously adjusted by means of proportional precision current-controlled reduction valves (15). These settings are selected individually, depending on the shaft’s structure, so that the supported journals experience no deflection, and that the ball-ended centres (3 and 4) are unloaded elements which fix the shaft in place. Ball-ended centres do function as the measuring base (relative to which the geometrical deviations of the measured shaft are determined). The articulated (multi-linked) mounting of the support head ensures constant contact between the rollers of the rolling V-block and the shaft journal, even when the support is placed on a base that is not entirely flat. The variable forces (depending on the crankshaft angle) are previously determined using the strength calculation software—FEM [53,54,55,56].

However, a change in the force values obtained by changing the pressure of the medium driving the support actuators does not ensure that the required relieving forces are unambiguously set. The actual forces exerted by the pressure-controlled supports depend on a number of factors that determines the characteristics of an actuator itself, such as friction resistance, type of driving medium, shape of the actuator elements, materials used and the actuator’s structural design. Thus, the system also uses force transducers (7) located between the heads (6) and the actuators (10) of the supports (5). In this way, the required force is the parameter that regulates the pressure in the control valves (15). FT-5367-4kN strain gauge transducers (7) are used to measure compressive and tensile forces. Changes in the ambient temperature are compensated for by the sensor’s electrical circuit. The view of the transducer and its basic technical data are shown in Appendix A.

The pressure in the support cylinders is thus controlled by the force and adjusted to the required relieving force. The system for measuring the geometric deviation of the shaft, which consists of the trolley (19) with a tripod (21), a measuring sensor (22) and a laser distance meter (20), moves along the shaft on precise and rigid guides (18), all parts of which are shown in Figure 5. The tripod with a sensor can slide and self-align and is mounted on the trolley carrying plate. In this way the sensor’s altitude and angle relative to the measured profile of the roundness of the main journal in the adopted reference system of measurement are precisely adjusted. The positioning accuracy of this system is 0.1 mm. Previous studies by the authors have shown that the applied accuracy of the slides does not exceed 1% of the accuracy of reproduction of the measured outline. With an exemplary deviation of shape equal to 0.01 μm, this is sufficient for practical applications. The sensor is connected to the computer via an interface (23); an inverter controls the speed of the engine that turns the shaft. The rotational motion from the engine (12) is transmitted by means of a flexible coupling (13) with a shaft relief mechanism.

**Figure 6:**
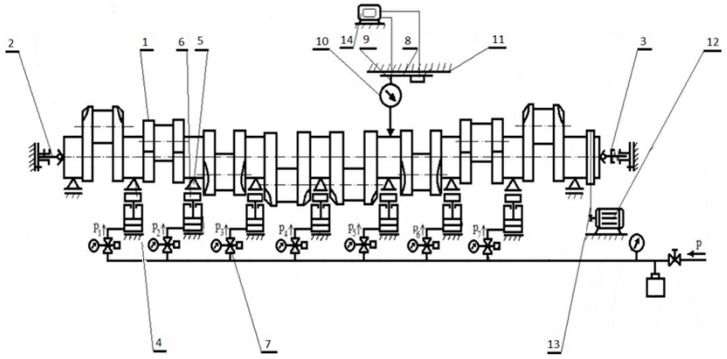
Diagram of the basic elements of the measurement system for a crankshaft fixed at the outermost journals in V-blocks [51].

As shown in Figure 5, the measuring system solution has been developed for cases where centre holes had previously been made in the shaft’s frontal faces. The measurement in this case corresponds to the conditions of non-reference measurements with the measured object being fixed in the centres, i.e., conditions of measurements similar to those provided on precise but expensive measuring machines. In the case where there are no centre holes in the shaft’s frontal faces, it is assumed that the shaft will be fixed with the outermost main journals on supports with fixed V-block heads and supported in the central part (as in the previous version of the measuring system) with a set of relieving supports. Such a solution is shown in Figure 6.

In this case, the external supports are the foundation responsible for the subsequent determination of geometric deviation. However, the pressures at the relief supports must be such that the heads (6) of the locating supports (5) with the outermost journals and at the same time they must not be overloaded. The axial displacement of the shaft is limited by the floating ball-ended centres (2 and 3), one of which is fixed, while the other exerts the axial clamping force, which ensures constant contact between the shaft and the centres. In this case, the conditions of measurements correspond to those of the reference measurements and the preparation of the measurement results must include a procedure for the transformation of the measured roundness profile into the so-called real transformed profile, developed in the case where the object measured is fixed in two V-blocks [44].

In the case of the reference measurements, which include measurements in V-blocks, there is a displacement of the object fixed in them. This displacement results from the fact that when the object is rotated, the successive points of the shaft’s main journals’ roundness profiles, which are usually irregular, continually make contact with the generators of the locating V-blocks. In line with this concept, a prototype of a system to eliminate any deformation of the crankshaft’s main journals during measurements of the shaft’s geometry was designed and built. One of the important elements of the flexible support system, which is responsible for the exertion of the set values of the reaction forces, is the control system. The correctness of the instantiation of the parameters set by the control system depends largely on the stabilization of the control signal.

## 5. Prototype Flexible Support Control System

### 5.1. Basic Assumptions

**Figure 7:**
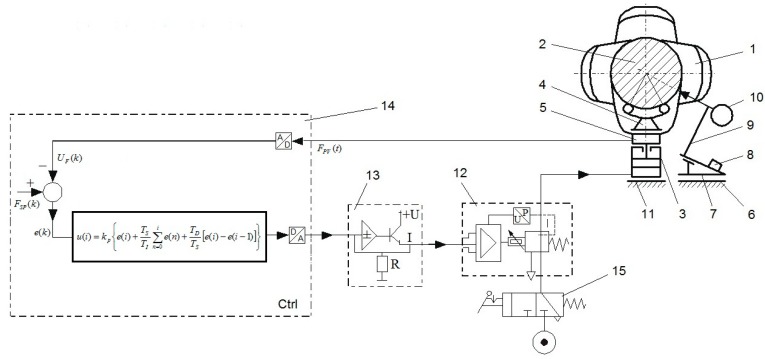
Basic elements of the flexible support control unit [50]: 1—crankshaft, 2—shaft’s main journal, 3—pneumatic actuator, 4—rolling, articulated, self-adjusting V-block head, 5—force sensor (force transducer), 6—guides, 7—trolley, 8—laser distance meter for measuring the longitudinal coordinate of the measured cross-section, 9—tripod, 10—sensor for measuring geometric deviations, 11—base, 12—proportional current-controlled reducing valve (controlled proportional regulating valve), 13—current relay, 14— programmable digital controller (control circuit), 15—feed valve, A—analogue signal, Ctrl—controller, D—digital signal, *e*(*k*)—an error signal (an input signal of the PID algorithm), *F_PV_*(*t*)—signal of realization force, *F_SP_*(*k*)—signal of the set force, I—current signal, *kp(i)*—proportional gain, P—pressure, R—resistance, *T_D_*—differentiation time, T_I_—integration time, T_S_—sampling time, U—voltage, *U_F_*(*k*)—signal corresponding with *F_PV_*(*t*).

A general scheme of the elastic support reaction force control unit is shown in Figure 7. The basic part of the control unit is the X20CP1586 control unit (B&R Industrial Automation, Eggelsberg, Austria), which is a programmable digital controller, typically used in industrial automation systems. The control device cooperates with the installation by means of input/output devices, i.e., digital/analogue and analogue/digital units.

The main task of the control unit is to provide the force FPV(t) that is stable and close to the set force FSP(t). A list of all the physical variables in the control system is given in Table 2. The input force FPV(t) (the reaction force applied where the head meets the main journal) is measured using a force transducer and sent to a control unit via the X20AI4632 analogue module (B&R Industrial Automation, Eggelsberg, Austria).

A pneumatic actuator with a PRE-I2 pressure controller (Hoerbriger, Zug, Switzerland), which operates as an analogue device, is used to exert the set reaction force. The analogue control signal U(t) activates the pneumatic actuator using the X20AO4622 analogue output module and pressure controller. The pneumatic actuator produces an effective manipulated signal, which is the force that is applied to the main journal of the crankshaft. The described algorithm for continuous PID control (proportional-integral-derivative) was used as an output to control the signal of the preset reaction force in the flexible crankshaft support system.

### 5.2. Filtration of the Signal from the Transducer

**Figure 8:**
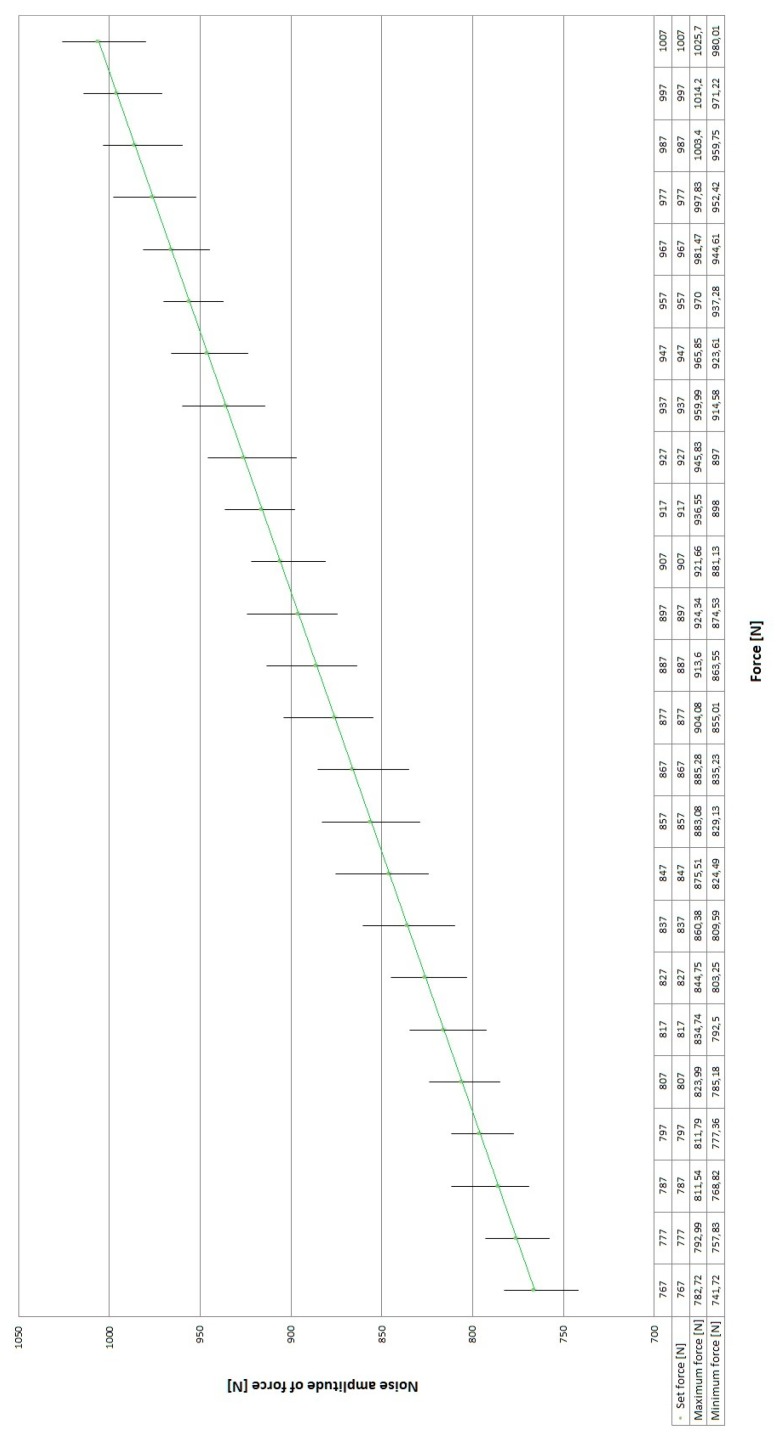
Noise amplitude values in the force signal from the transducer of the prototype system without filtration.

The signal obtained directly from the force transducer is relatively noisy, which results from the high sensitivity of the measuring instrument and its sensitivity to environmental influences (vibration). This problem is presented in Figure 8. The average amplitude with the force changing with increments of 10 N in the range 767 ÷ 1007 N was equal to 40 ÷ 60 N.

**Figure 9:**
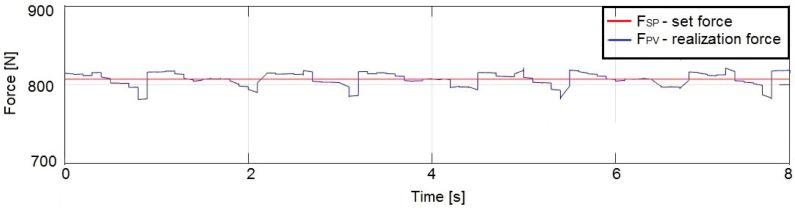
Experimentally obtained graph of the actuator output force for a setpoint value of 810 N without signal filtration in the control system.

The force changes with respect to an example setpoint of 810 N for an unfiltered signal (before noise removal) are shown in Figure 9.

From a practical point of view, the use of force transducers under industrial conditions with such behaviour may be considered as typical and not relevant for the achievement of the specified goal. The high sensitivity of the strain gauges used in force transducers leads to measurement signal fluctuations, followed by control signal fluctuations and then the heavy-duty operation of actuators, i.e., the need to act frequent.

**Figure 10:**
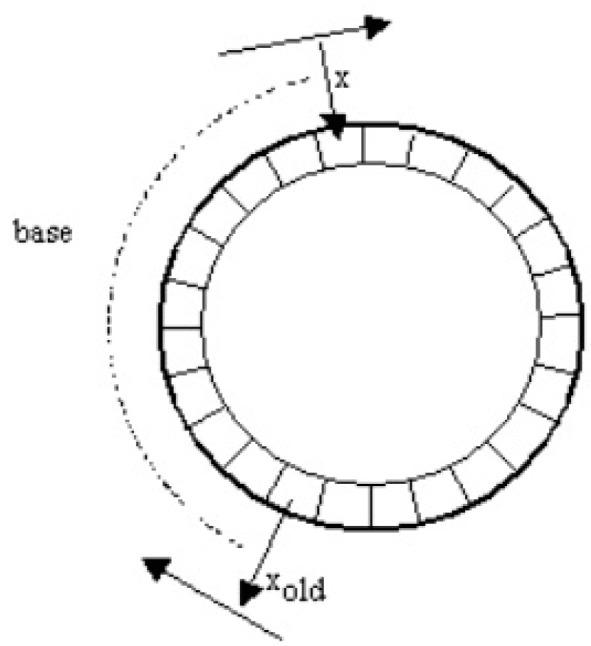
General concept of the moving average filter [58].

Considering the use of this type of transducer to exert the preset force in this control system, it was necessary to minimize the fluctuations of the control signal. For this reason, an additional filtering module was used in the force control algorithm; a moving average filter was used, the concept of which is shown in Figure 10.

The filter generates a constantly updated average of the signal from the input *x* using the previous number of cycles specified in the database and outputs it to *y* [58,59], according to the formula:

(1) $$\left\{ \begin{array}{l} y=\frac{s_{old}-x_{old}+x}{b}\\ s_{old}:=s_{old}-x_{old}+x \end{array}\right.,$$

where:

*s_old_*—sum of the samples of the signal *x*,
*b*—number of samples of the signal *x* to be summed (averaging base),
*x*—value of the current signal sample,
*x_old_*—value of the historical sample at a distance equal to the base
*y*—averaged value of the signal *x*.

**Figure 11:**
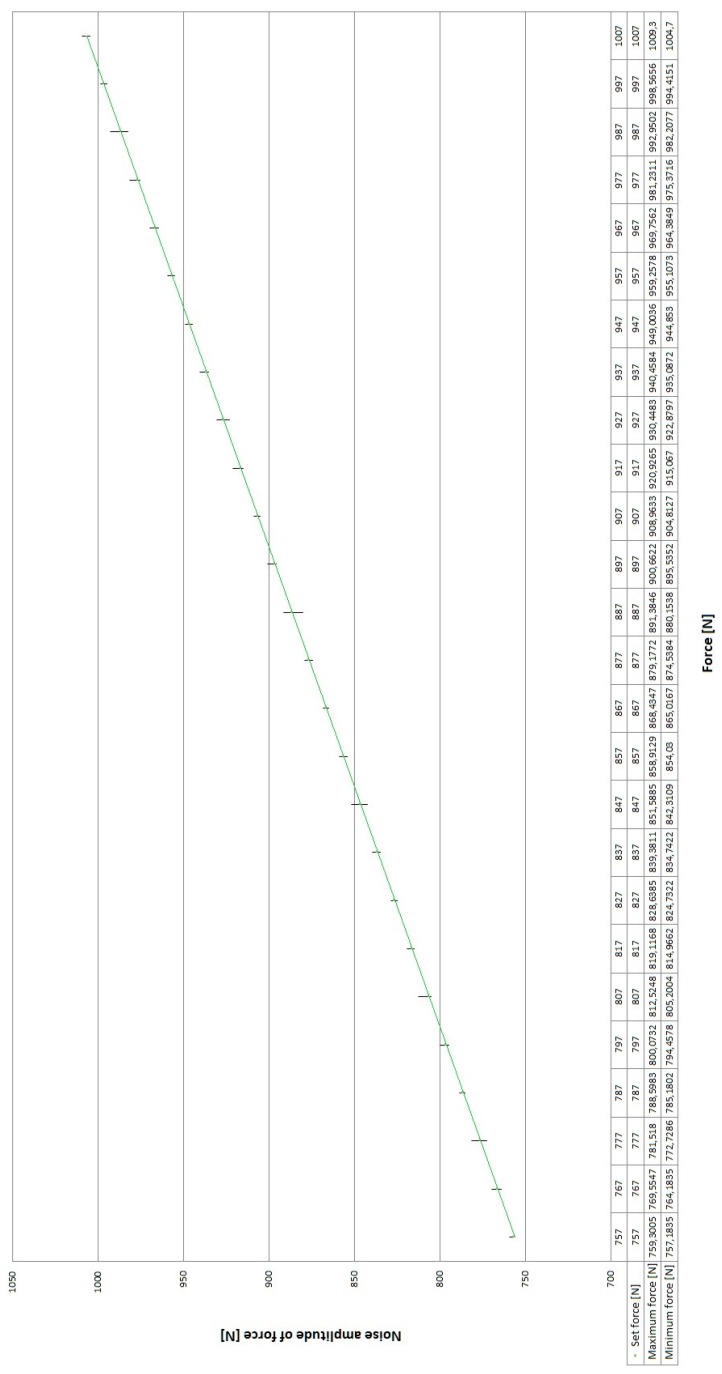
Amplitude of the noise in the force signal from the transducer for the prototype system with a moving average filter for force increments of 10 N in the range from 757 N to 1007 N.

Figure 11 shows the filtered signal with a force changed by increments of 10 N, in the range from 757 N to 1007 N, using a moving average filter.

**Figure 12:**
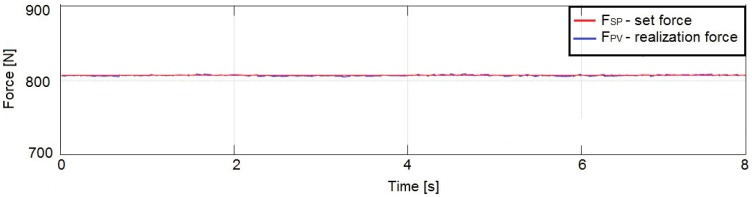
Experimentally obtained graph of the actuator output force for a setpoint of 810 N using a moving average filter in the control system.

The changes in the force with respect to an example setpoint of 810 N for a filtered signal (noise removed using a moving average filter) are shown in Figure 12.

The conducted experiments show that, due to use of the filter, the average noise amplitude decreased to 4–5 N, which can be considered as satisfactory for the completion of the task that has been undertaken.

### 5.3. Selection of the Operating Parameters of the Controller

From the perspective of the control of the preset value of the reaction forces, the appropriate dynamics of the control unit is important, i.e., the rate of the setting system’s response to the preset impulse of the actuator (the former is a pneumatic actuator; the latter is a precise current-controlled valve). The PID controller is used [60,61].

In order for the task to be performed, it is important that the control signal changes with minimal overshoot [58]. This reduces the influence of the dynamic effect of the control unit components on the measurement of geometric deviation in the crankshafts. From a practical point of view it is also advisable to minimise the value of overshoot. In this case, the risk of exceeding the permissible technological limitations of the actuators and those of the object being controlled is lower, as is the possibility of system failure or its complete destruction.

With this in mind, a number of tests were carried out in order to determine the dynamic characteristics of the control unit with quality indicators that were most favourable for the completion of the task undertaken, thanks to which, and without losing the dynamic properties of the unit, the response time to the preset impulse of the actuator would be the shortest.

**Figure 13:**
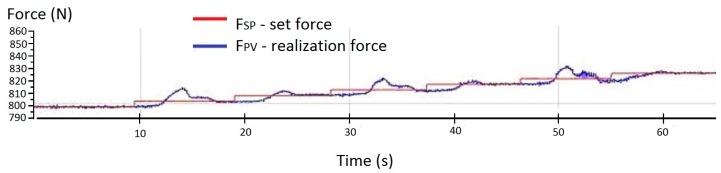
Experimentally obtained characteristics of the dynamics of the control unit’s operation with the preset reaction force being changed in steps of 5 N for incorrectly selected controller settings.

The characteristics of the dynamics of the control unit’s operation with the preset force being incremented by 5 N is presented in Figure 13. The distinctive elements of the characteristics are visible overshoot for every change in the preset force. The controller operation is critical to the sensing experiment. In the presented case, each change of the preset force value causes an overshoot equal to 1 ÷ 7 N, which in relation to the preset force value constitutes a relative value within the range 0.12 ÷ 0.87%. The adjustment time for the presented test ranges from 5 ÷ 8 s.

**Figure 14:**
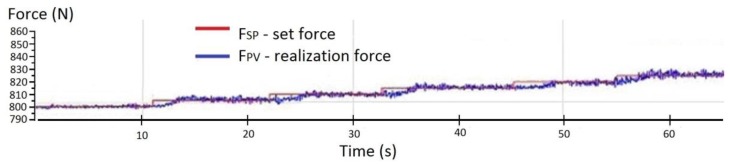
Experimentally obtained statistics on the dynamics of the control unit’s operation with the preset reaction forces being changed by 5 N for a system with improved control quality.

Figure 14 shows the characteristics of the dynamics of the control system’s operation with the preset reaction force being changed by 5 N, for the experimentally corrected control system settings. In the presented case, each change of the preset force value causes an overshoot equal to 1 ÷ 2 N, which in relation to the preset force value constitutes a relative value within the range of 0.12 ÷ 0.24%. The adjustment time for the presented test ranges from 3 ÷ 4 s, which indicates that a very high-quality control process has been performed.

The research that has been conducted, some results of which have been presented in this part of the article, has resulted in the determination of the most favourable parameters of the signal controlling the set value of the reaction force in the flexible crankshaft support system. One of the important advantages of the control unit is its gentle and stable characteristic of operation dynamics and, as a result, the negligible influence of the support conditions on the measurement of the geometry.

## 6. Results and Discussion

### 6.1. Preliminary Calculations

**Figure 15:**
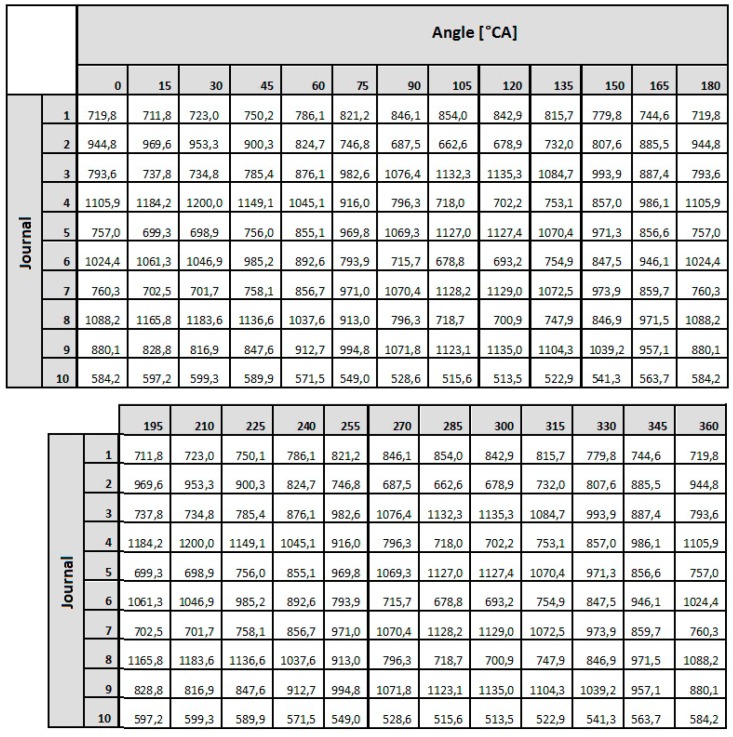
The required values of the reaction forces at the journals to guarantee zero deflection when the shaft’s rotation angle is changed in steps of 15°—calculations performed with Nastran FX 2010.

**Figure 16:**
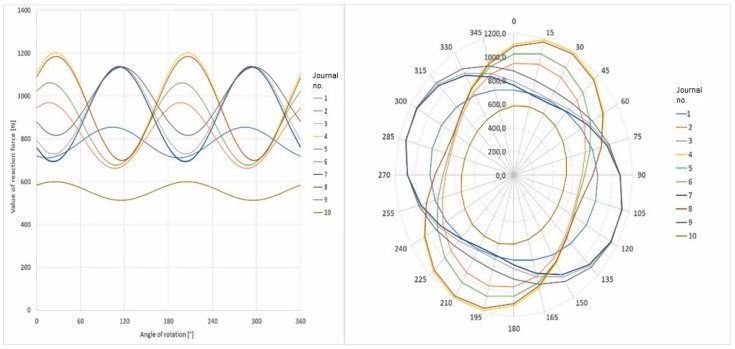
Distribution of the reaction forces that guarantee zero deflection of the main journals for a crankshaft with a diameter of the main journals being 149 mm and crankpins of 114 mm, and with oval crank webs with dimensions of 252 × 358 mm [63].

The procedures that have been presented to determine the required values of the reaction forces that ensure that the shaft’s elastic deflection have been eliminated were applied in the measurements of the crankshaft’s geometric deviation. These were carried out with the measuring system developed by the Maritime University in Szczecin. The first step was to determine the forces that result in the elimination of the deflection of the shaft whose geometry is being measured. Sample results of the calculations of the forces ensuring the elimination of any deflection at the shaft’s journals, carried out for the object selected for testing, that is the crankshaft of the main propulsion medium-speed engine of the Buckau Wolf R8 DV136 ship mentioned above, have been presented in Figure 15. The crankshaft used has a length of 3630 mm and a weight of 9360 N and is fitted with ten main journals, each are 149 mm in diameter and it has eight crank pins, each 114 mm in diameter. Figure 15 shows the required values of the reaction forces at the main journals, which guarantee zero deflection at the journals when the shaft’s rotation angle changes in steps of 15°. The forces were calculated using the MES Nastran FX 2010 strength calculation software. A graphical presentation of the results obtained is shown in Figure 16.

The analysis of the results obtained showed that the distribution of the forces guaranteeing zero deflection on the individual journals for a shaft rotation angle ranging from 0 to 360° and presented in the polar coordinate system, created an image of a regular ellipse, in which the Cartesian coordinate system corresponds to a function similar to a cosine curve. The mathematical description of this function is based on the criteria of harmonic analysis [62]. According to this tool, the main component of the mathematical profile will be the second harmonic component reflecting the ovality of this profile. This was confirmed by calculations carried out in the MATLAB environment (MathWorks, Natick, MA, USA).

Harmonic components were calculated on the basis of the functional dependency that is given by the following formula [63]:

(2) $$R\left(\phi \right)=R_{0}+\sum_{n=1}^{k}C_{Rn}\cos n\left(\phi -\phi_{R}{}_{n}\right),$$

where:

*R_o_*—averaged value of the calculated reaction force,
*C_Rn_*—amplitude of the successive, *n*-th harmonic component of the reaction force function,
*φ_Rn_*—phase shift of the successive *n*-th harmonic component of the reaction force function.

The dependency (2) can be presented alternatively in the form of [64]:

(3) $$R\left(\phi \right)=R_{0}+\sum_{n=1}^{k}A_{Rn}\cos n\phi +\sum_{n=1}^{k}B_{Rn}\sin n\phi ,$$

where: *A_Rn_*, *B_Rn_*—the coefficients of the amplitudes of successive *n*-th harmonic components of the reaction force function.

**Figure 17:**
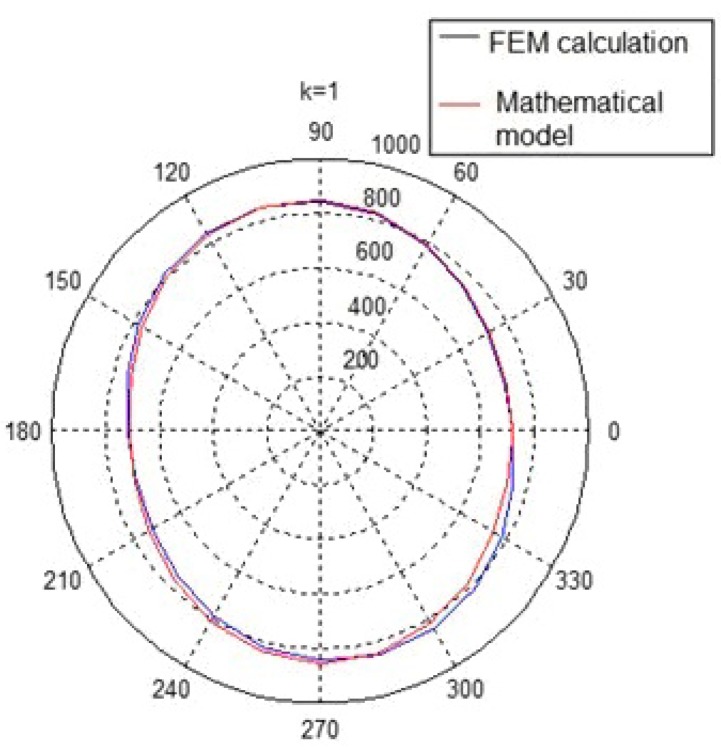
Distribution of the forces ensuring zero deflection at journal no. 1 (counting from the timing gear) in the polar coordinate system [64].

The preset values of the reaction forces calculated with the use of the strength calculation software were then replaced by a mathematical expression, that is, a trigonometric Fourier series. That expression enabled the continuous computer-aided monitoring of the operation of the precisely controlled valves, which adjusted the supply of the medium driving the flexible relief supports. Figure 17 shows the forces eliminating any deflection for a selected journal of the crankshaft in both the Cartesian and polar coordinate systems. The distribution of the forces obtained by the strength calculations and the mathematical model for a full revolution of the crankshaft has been presented. The graphical record of force changes ensuring zero deflections on the pins was created based on strength calculations with a change in the shaft rotation angle every 15°. To control the set value of the reaction force in a continuous manner (for any angular position of the shaft), it was advisable to replace such a recording form with a mathematical model.

Determination of the values of the reaction forces for any angular position of the shaft is important for the proposed measurement method. These reaction forces are calculated using FEM. Therefore verification of the correlation between analytical model and FEM values is important. In order to verify the usefulness of the models based on the analytical notation, it was then evaluated as to whether the force charts obtained by the strength calculations were consistent with the mathematical notation in the form of a trigonometric Fourier series. The comparative assessment was made by calculating the so-called mutual correlation factor [65,66,67], described by the relation:

(4) $$\rho (\gamma_{\phi })=\frac{2\int_{0}^{2\pi }r_{1}(\phi )r_{2}(\phi +\gamma_{\phi })d\phi }{\int_{0}^{2\pi }r_{1}{\left(\phi \right)}^{2}d\phi +\int_{0}^{2\pi }r_{2}{\left(\phi \right)}^{2}d\phi },$$

where:

*r*_1_*(φ)—*the chart obtained by means of the strength calculations,
*r*_2_*(φ)*—the charts obtained using mathematical notation,
$\gamma_{\phi }$—the phase shift between the charts being compared.

The calculated values of the mutual correlation factors for the group of tested journals have been presented in Table 3.

For this example, the mutual correlation coefficients for the journals were between 0.9542 to 0.9578, which, according to the evaluation scale [68] adopted by Guilford to determine the degree of correlation, indicated a very high correlation.

### 6.2. Experimental Verification of the Elimination of Crankshaft Deflection

**Figure 18:**
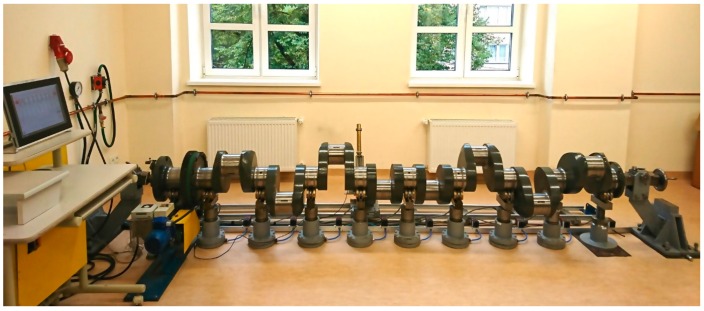
A prototype test bench developed at the Maritime University in Szczecin for the measurement of the geometric deviation in large-size crankshafts equipped with a flexible support system.

The testing aimed at verifying the performance of the proposed operating system was carried out on a testing bench at the Maritime University in Szczecin, which was developed in accordance with the previously presented concept. The system provided a flexible support for the measured crankshaft, a view of the system is presented in Figure 18.

The tests covered the measurements of the geometric deviation of the main journals of the crankshaft of a Buckau Wolf R8DW 136 engine, for which the values of the reaction forces ensuring zero deflection at the main journals were calculated. The geometric deviation in the shape profiles were measured using two different measurement systems, namely, a measurement system with the flexible support of the measured object, fixed at the outermost faces by means of centres and a measurement system whose measurement results were taken as a reference.

**Figure 19:**
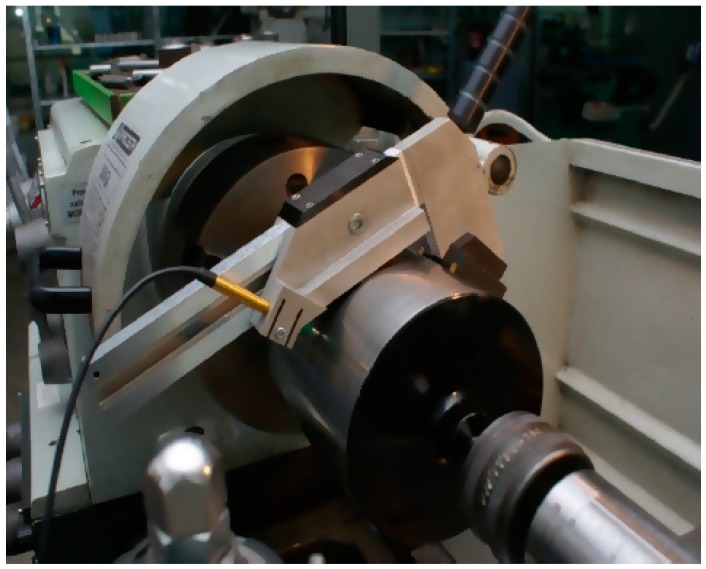
View of the MUK 25 ÷ 600 measuring head [50].

The standard measurements were performed with the use of a measuring system with a MUK 25 ÷ 600 head (Figure 19) and the SAJD software [12,44] operating under industrial conditions.

The system, which has been adopted as a model, was designed for reference measurements of the roundness profiles of cylindrical surfaces. The MUK 25 ÷ 600 head was directly mounted on the surface of the tested journal during the measurements, as a result of which the assessment of the shape profile was not dependent on the conditions of the supports of the object being measured [69]. The selection of this system for standard measurements was also favourable, because the results of the measurements made with the use of the compared systems could be evaluated with a similar mathematical apparatus based on the harmonic analysis of the measured profiles.

In general, the measurements of an object fixed in V-blocks or centres consist of gauging the composition of the shape deviation and axis position in successive cross-sections of a set of journals of a rotating crankshaft. The centre of a section profile being measured may move around the axis of rotation determined by the measuring system when the shaft is fixed in V-blocks or relative to the axis determined by the centres when the shaft is fixed in centres. The measurement results contain a description of the profile of the shape of the measured cross-section and the eccentricity representing the position of the centre of the profile of the section measured in relation to the axis of rotation that was determined by the centres. This measurement method requires the results to be processed in two stages, the first of which allows for the determination of any deviation in the shape. The second allows any deviation in the position of the axis to be determined. It was assumed that in both stages, the reference element was the root mean square. The mean square element was adopted as a reference to assess any deviation in the shape and position of the axis due to a number of advantages of this type of element, as demonstrated in the literature [12,44,70,71]. The shape profile was determined to be a root-mean-square circle obtained from the measured shape. With respect to the coaxiality evaluation, the axis was obtained from the previously found centres of the particular shape profiles measured at specific cross-sections along the shaft. The applied harmonic analysis of the roundness profiles can be used to present the profiles measured in this way as a sum of the terms of a trigonometric Fourier series e.g., in the form of a finite sine transform—similarly as in Equations (3) and (4) [63].

**Figure 20:**
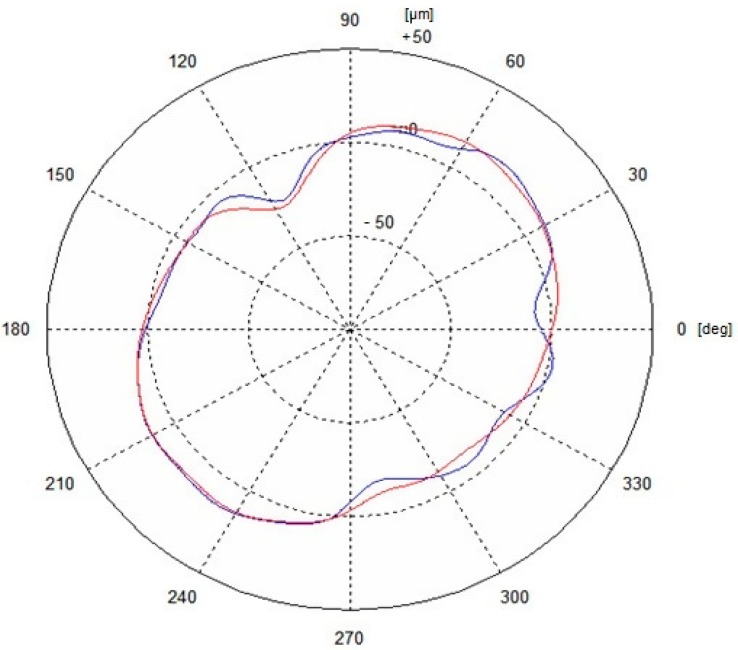
Comparison of the profiles measured with the prototype system (**blue**) and with the model system (**red**) of the no. 7 journal of the tested crankshaft [67].

The measurements adopted were carried out in the polar coordinate system. The measured values at the individual cross-sections l_i_ were the values of the change in the radius r_ji_ for a given rotation angle of the shaft φ_ji_. Considering that the shaft rotates by a constant angle φ_ji_, according to the literature [8,52], the coordinates x_j_ and y_j_ of the centre of any roundness profile (located at a distance l_i_) in relation to the axis of rotation realized by the fixing centres can be determined from the relationships (8) and (9), taking n = 1. In the meaning of the roundness profile expressed by a trigonometric Fourier series, these coordinates reflect the extent to which the profile is eccentric in relation to the measurement base, which in this case is the axis determined by the locating centres. Figure 20 shows the overlapping roundness profiles that were obtained from both the prototype system and the model system.

The corresponding roundness profiles, the model one *r*_1_(*φ*) and the tested one *r*_2_(*φ*), which were prefiltered with respect to the harmonics n = 2~15 were subjected to comparative evaluation with the use of the standardized mutual correlation function given by the formula (4). Determination of the values of the reaction forces for any angular position of the shaft is important for the proposed measurement method. These reaction forces are calculated using FEM. Therefore verification of the correlation between experimental and FEM values is important. Examples of the values of the roundness determined for the no. 7 main journal of the measured crankshaft, obtained from the results of the measurements made with the tested measuring system (Δ*R_z_*) and the model system (Δ*R_w_*) as well as the coefficient of correlation between the compared profiles ρ, are presented in Table 4.

The mathematical apparatus used in the preparation of the measurement results was similar to the one used in recording the changes in the reaction forces guaranteeing the elimination of any shaft deflection, which means it could be used to solve a wide range of problems. The results of the testing showed a very high degree of similarity of the measured roundness deviation and a high degree of correlation between the compared profiles. The calculated value of the mutual correlation coefficient for the compared profiles was ρ = 0.9662, which corresponds to a very good fitting. In order to verify that the system functions correctly, the transversal accuracy of the slides was analyzed, a portion of which is presented in Table 5.

The obtained sample values of the method’s experimental measurement error determined for the measured roundness deviation (Table 5), ranged from 7.9–10.9%. According to Adamczak [65] and Nowicki [72], the recommendations concerning the accepted ranges of the method’s accuracy when used to measure the geometric structure of surfaces are presented in Table 6.

When comparing the results presented in Table 5 against the recommendations described in Table 6, it may be assumed that the experimental error of the method can be used to sufficiently measure its accuracy. The results show that the accuracy of the proposed measurement system fully qualifies the method to a group of methods that may be used in industrial measurements or scientific research.

## 7. Conclusions

The results of the tests that were carried out have led to a conclusion that the proposed method for the controlled implementation of the required variable reaction forces of the supports effectively eliminated the deflection and elastic deformation of a crankshaft under the influence of its own weight. This has provided the basis for the further conclusion that the values of the geometric deviation, determined on the basis of the proposed measuring system, were correctly determined values with a high degree of correspondence to their real values.

Taking into account the properties of the proposed system, it can be assumed that such a system, when appropriately validated, could be useful for the complete measurement of geometric deviation in large crankshafts carried out in industrial conditions, as well as repair docks and workshops dealing with repairs of marine engines.

The paper shows that the methods currently used to measure the geometry of large-size shafts are subject to significant errors related to elastic deformation of the shaft. Alternative methods do not solve this problem. The shafts described in the article are used mainly in slow-running, high-power engines, which are currently produced only by three manufacturers, and 80% of the global production belongs to MAN. The shafts of the mentioned engines are available in several base versions, so the method proposed by the authors can be successfully applied in practice, assuming that several FEM models for the mentioned shafts are developed.

It should be emphasized that one of the essential features of the system presented in the article is its universality. The number and location of pneumatic cylinders can be adjusted to suit any crankshaft design.

The presented system will be further developed to improve the user interface [73], to use new construction materials for prism rollers, cylinder heads, cylinder stands, and lightweight and durable composite brackets [74,75].

## 8. Patents

Nozdrzykowski, K. Device for measuring positional deviation of axis of crankshaft pivot set. Polish Patent Office, PL393829-A1; PL218653-B1.

## Figures and Tables

**Table 1:** Methods and techniques used to measure shafts, broken down by their dimensions and the position of an axis during measurement.

Methods of Measurement		Crankshaft Type					
		Small		Medium		Large	
		Positioning of the Shaft Axis during Measurement					
		Horizontally	Vertically	Horizontally	Vertically	Horizontally	Vertically
non-reference	in centres	W	-	-	-	-	-
	in a device: – with a rotary spindle – with a rotary table	-	S	-	S	-	-
		-	S	-	S	-	-
reference	in 2 V-blocks	W	-	-	-	-	-
	in 4 V-blocks	-	-	W	-	-	-
	in *n* V-blocks	-	-	-	-	W	-
Others: – scanning – photometry	in *n* V-blocks	-	-	-	-	S	-

W: measurements that do not require special tooling (carried out in workshop conditions); S: measurements carried out with special measuring machines; –: not used.

**Table 2:** Specifications of the FT-5367-4kN transducer [57].

Signal	Description	Range	Unit
*F_SP_ (t)*	The value corresponding to the set force	0–2000	N
*F_PV_ (t)*	The value corresponding to the current force.	0–2000	N
*P_CV_(t)*	The value corresponding to the pressure signal	0–200	kPa

**Table 3:** The calculated values of the mutual correlation factors for the tested journals [64].

Journal Number	Mutual Correlation Factor *ρ*(*φ*)
1	0.9578
2	0.9568
3	0.9548
4	0.9543
5	0.9549
6	0.9562
7	0.9549
8	0.9542
9	0.9542
10	0.9544

**Table 4:** The roundness deviation for the example of the no. 7 main journal of the measured crankshaft, obtained from measurements made with the prototype measuring system (Δ*R_z_*) and the model system (Δ*R_w_*).

Journal Number	Deviation in Roundness Δ*R_z_* [μm]	Deviation in Roundness Δ*R_w_* [μm]	Correlation Factor *ρ* [-]
7	42.682	42.038	0.9662

**Table 5:** Sample results of statistical investigations into the experimental value of the relative error of the method of determining the roundness deviation.

No.	Analysed Parameter of Relative Error of the Method		Sample Name		
			Journal 4	Journal 5	Collective Sample
1	Sample size *n_s_*		20	20	40
2	Observable value—relative measurement error *w*_Δ*R*_	*w*_Δ*R*max_	0.056670	0.070230	0.070230
		*w*_Δ*R*min_	–0.06764	–0.09030	–0.09030
3	Mean value of the experimental measurement error		0.0022845	–0.015136	–0.006426
4	Confidence interval for the mean error value of the measurement at β = 0.95		0.0022845 ± 0.01736	–0.015136 ± 0.02098	–0.006426 ± 0.01330
5	Significance test for average value measurement error		Drop *H*_0_ in favour of *H*_1_	Drop *H*_0_ in favour of *H*_1_	Drop *H*_0_ in favour of *H*_1_
6	Variation in sample *s*^2^ for error		0.0013776	0.0020145	0.001730
7	Mean deviation *s* for error		0.0371160	0.0448842	0.041599
8	Significance test for variance of method error		Drop *H*_0_ in favour of *H*_1_	Drop *H*_0_ in favour of *H*_1_	Drop *H*_0_ in favour of *H*_1_
9	Confidence interval for a single measurement error at β = 0.95		0.0022845 ± 0.07760	–0.015136 ± 0.09380	–0.006426 ± 0.08415
10	Measurement accuracy of the DPM method		7.9%	10.9%	9.06%

**Table 6:** Accepted ranges of the method’s measurement accuracy when used to measure the geometric structure of the surface [65,72].

Measuring Accuracy of the Method in %	Type of Measurement
2–5	Measurements of roughness, surface waveformity, and shape outline standards and basic research
5–15	Scientific research
10–25	Technical inspection and industrial measurements

**Figure A1:**
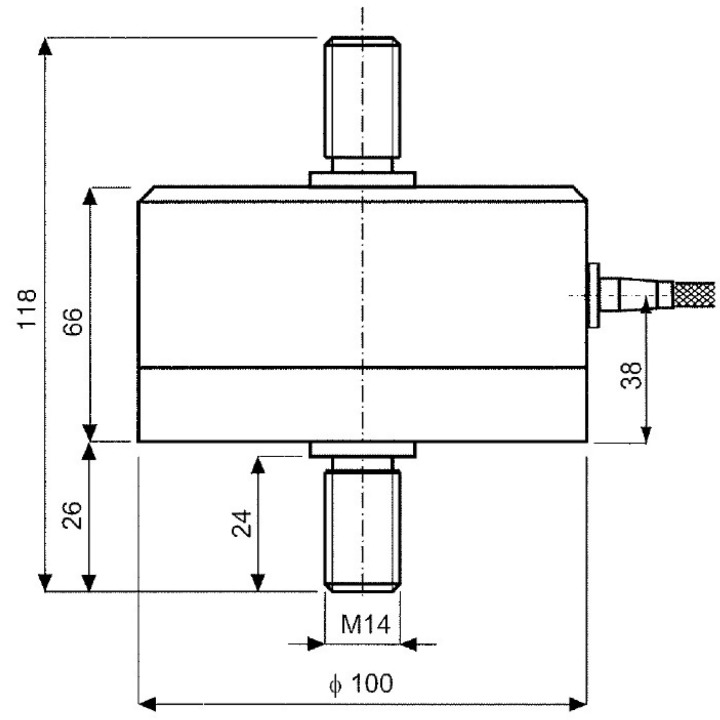
Strain gauge force transducer, type FT-5367-4 kN [57].

## Appendix A

**Table A1:** Specifications of the transducer, FT-5367-4kN [57].

Specific Characteristic	Description
Material of the bearing elements	Stainless steel 1.4057
Measuring range	0 ÷ ±4 kN
Measuring overload	1.25 × measuring range
Strength overload capacity	2.5 × measuring range
Supply voltages	24 V DC (12 ~ 30 V DC)
Output signal	0 ÷ ±5 V
Accuracy class	1% (acc. to PN-EN ISO 7500-1)
Tolerance of zero signal	±1%
Temperature instability	±0.01%/K
Ambient temperature range	−25 ÷ +45 °C
Degree of protection	IP68
Output cable	3 × 0.50 mm^2^, shielded
Cable length	6 m
